# Astragaloside IV Exerts Cardioprotection in Animal Models of Viral Myocarditis: A Preclinical Systematic Review and Meta-Analysis

**DOI:** 10.3389/fphar.2019.01388

**Published:** 2019-11-28

**Authors:** Zhuang Zhuang, Zi-Hao Wang, Li-Hui Deng, Qun Zheng, Guo-Qing Zheng, Yan Wang

**Affiliations:** ^1^Department of Cardiology, the Second Affiliated Hospital and Yuying Children’s Hospital of Wenzhou Medical University, Wenzhou, China; ^2^Department of Integrative Medicine of Neurology, the Second Affiliated Hospital and Yuying Children’s Hospital of Wenzhou Medical University, Wenzhou, China

**Keywords:** Astragaloside IV, myocarditis, efficacy, mechanisms, meta-analysis

## Abstract

Astragaloside IV (AS-IV), the essential active component of astragalus, possesses diverse biological activities that have beneficial effects against cardiovascular disease. Here, we conducted a preclinical systematic review of 15 studies including 577 animals to establish the efficacy and potential mechanisms of AS-IV for animal models of viral myocarditis (VM). Six databases were searched from inception to October 2018. Application of the Cochrane Collaboration’s tool 10-item checklist and Rev-Man 5.3 software to analyze risk of bias of studies and data on outcome measures revealed study quality scores ranging from 2 to 5. Compared with the control group, AS-IV induced a marked decrease in mortality (P < 0.05), inflammation of myocardium and pathological score (P< 0.05) and cardiac enzymes expression (P< 0.05), and improved the function of the heart (P< 0.05). The potential mechanisms of AS-IV action were determined as anti-remodeling of myocardium (n = 1), anti-virus (n = 2), antioxidant (n = 2), anti-inflammatory (n = 6), anti-apoptosis (n = 1) and alleviation of myocardial fibrosis (n = 2). The collective results indicate that AS-IV exerts cardioprotective effects in animals with VM *via* multiple signaling pathways.

## Introduction

Myocarditis is pathologically and clinically defined as an inflammatory disease of the heart muscle based on histological, immunological, and immunohistochemical criteria of cardiomyopathy classification from the World Health Organization (WHO) (Pollack et al., 2015). Myocarditis often arises from infection with common viruses, such as respiratory viruses and enteroviruses, and is most commonly associated with coxsackievirus B (Dennert et al., 2008). Myocarditis infection can be subdivided into three pathological stages (Mason, 2003). In the first stage, viral-mediated lysis directly destroys cardiomyocytes, leading to cardiomyocyte damage and cardiac dilation (McManus et al., 1993). The second stage is imbalance of immune regulation resulting from myocardial cell damage (Lawson, 2000). Finally, the typical manifestation of dilated cardiomyopathy (DCM) occurs due to extensive myocardial injury (Caforio et al., 2002). Viral myocarditis (VM) has the characteristics of myocardial inflammation, often developing into chronic dilated cardiomyopathy, acute myocarditis and even congestive heart failure (Kühl and Schultheiss, 2009). VM accounts for 12% of sudden deaths in patients under 40 years of age and is the leading cause of dilated cardiomyopathy, resulting in 50% of patients deaths within 1–2 years after diagnosis (Cooper et al., 2014). The incidence of myocarditis worldwide was reported a 22 cases per 100,000 individuals in 2013, and about 1.5 million patients were diagnosed with myocarditis according to the ninth revised diagnosis of the International Classification of Diseases (GBD 2015 Disease and Injury Incidence and Prevalence Collaborators, 2016). For VM, antiviral drugs, physical activity restriction and myocardial nutrition are the main routine clinical treatments. Patients should follow the current guidelines for heart failure treatment in cases of left ventricular dysfunction or symptomatic heart failure (Ponikowski et al., 2016). However, establishing the potential benefits of immunomodulators and antiviral therapy is currently at the preliminary research stage (Pollack et al., 2015).

AS-IV, the essential active component of astragalus, has been traditionally used to treat various diseases. This compound is frequently reported to exert protective effects on cardiovascular, nervous and immune systems due to its antioxidant and activitiesanti-apoptotic activities as well as regulatory effects on calcium balance (Ren et al., 2013). Systematic studies on animal models play an important role in drug research and provide substantial evidence to support application in the clinic (Sena et al., 2014). The purposes of the present study was to investigate the efficacy and potential mechanisms of action of AS-IV in animal models of VM.

## Methods

The study was carried out according to the Preferred Reporting Items for Systematic Review and MetaAnalyses (Stewart et al., 2015).

### Search Strategies

Based on the literature retrieval from PubMed, Wanfang database, EMBASE, China National Knowledge Infrastructure (CNKI), Cochrane Library and VIP databases, animal experimental studies of AS-IV for VM were identified. All search strategies were performed from inception until October 2018. We selected the following terms: 1. Astragaloside; 2. Myocarditis; 3. 1 AND 2; 4. Animals NOT humans; 5. 3 AND 4.

### Eligibility Criteria

Studies that met the following criteria were included: (1) animal models of VM were established through various ways, (2) treatment groups were administered any dose of AS-IV only, and comparators given non-functional and isasteric liquid (carboxymethyl cellulose or normal saline) or no treatment, and (3) the primary outcome measures were mortality rate, histopathological changes of myocardium, indicators of cardiac ultrasound, cardiac troponin, cardiac enzymes and/or level of ST-segment depression. Cardioprotective mechanisms of AS-IV action against VM were used as the secondary outcome measure. Exclusion criteria were as follows: (1) duplicate publication, (2) not *in vivo* studies, (3) comparison with other traditional Chinese medicine (TCM), (4) combinations with other medicine, and (5) lack of a control group.

### Data Extraction

Two authors independently extracted the following details: (1) name of the first author and publication year, (2) characteristics (species, number, sex, and weight) of the animals used in each study, (3) methods to establish animal models of VM, (4) the therapeutic regimens of treatment and control groups, including method of administration, therapeutic drug dosage and sessions of treatment, (5) primary and secondary outcomes. If the results were obtained at different time-points or following administration of different doses of drug, only the final measured value and data obtained with the highest dose of drug were included. We attempted to contact authors for more information when published data for some records were only shown in a graphical format. In cases where no response was received, data in the graph were measured.

### Risk of Bias in Individual Studies

The risk of bias was assessed by two independent authors through applying the ten-item scale (Hooijmans et al., 2014), with minor modifications. Risk of bias of the following domains were assessed: A: sequence generation; B: baseline characteristics; C: allocation concealment; D: random housing and other animal welfare; E: blinding of caregivers and/or investigators; F: random outcome assessment; G: blinding of outcome assessor; H: complete outcome data; I: selective outcome reporting; J: other sources of bias. One point was awarded for each item. Divergent findings between the two authors were settled through resolving by consensus or arbitration by the corresponding author.

### Statistical Analysis

RevMan version 5.3 was utilized for statistical analysis. Standardized mean difference (SMD) instead of Mean differences (MDs) was employed as a summary statistic when data were not reported on the same scale. Heterogeneity and choice of effects models were investigated with the aid of standard chi-square test and I² statistic test. Differences were considered significant at P values <0.05.

## Results

### Study Selection

**Figure 1:**
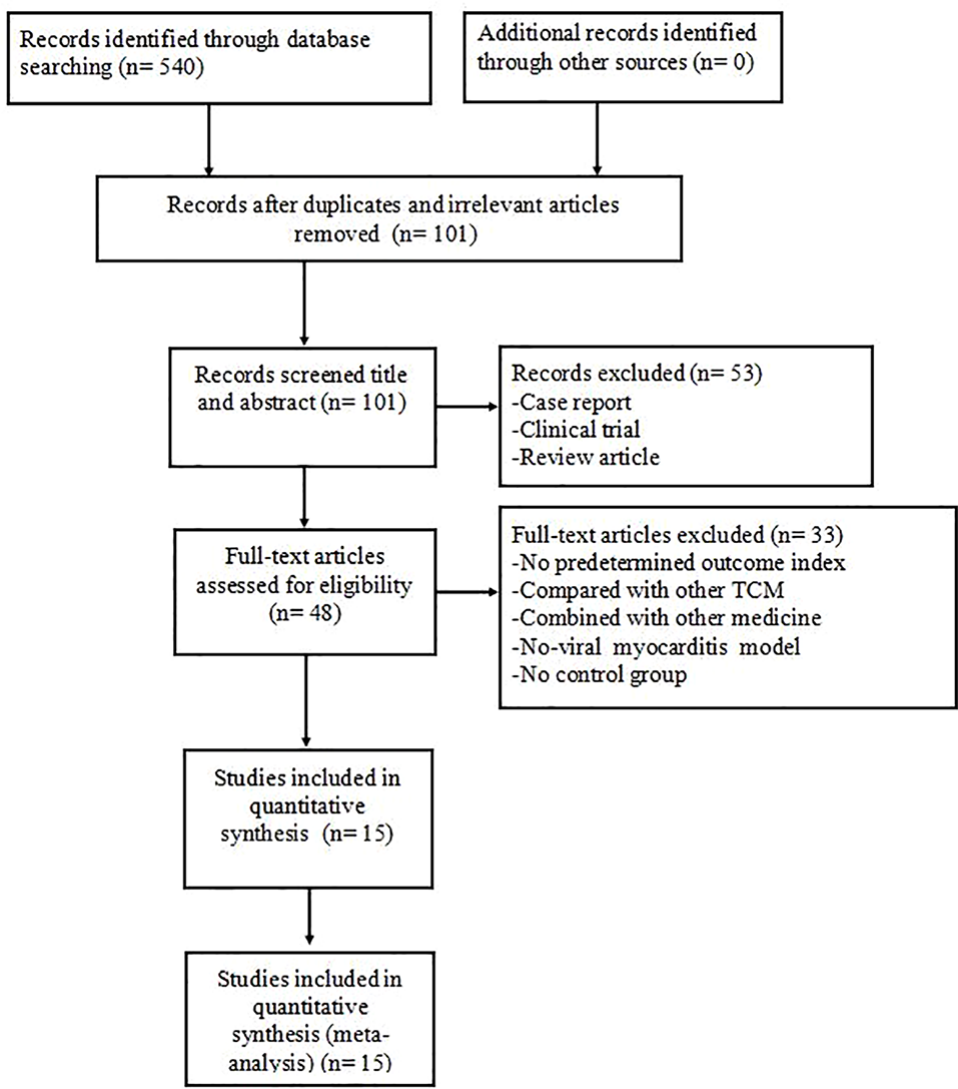
Summary of the process for identifying candidate studies.

We identified a total of 540 hints on the basis of pertinent literature retrieval from the databases. After removing 439 reduplicated or irrelevant articles, 101 reports remained. Next, we excluded 53 non-animal studies through screening the titles and abstracts. Overall, 33 articles were excluded after reading the remaining full-text articles owing to: (1) no predetermined outcome index, (2) comparison with other TCM, (3) combination with other medicine, (4) non-viral myocarditis models, and (5) lack of a control group, leading to the final selection of 15 eligible articles (Zhang et al., 2003; Yu et al., 2005; Zhang et al., 2006; Wang and Li, 2007; Luo et al., 2008; Liu et al., 2009; Luo et al., 2010; Chen et al., 2011; He and Li, 2011; Wang, 2012; Zhou et al., 2012; Liu et al., 2014; Gui et al., 2015; Tian et al., 2015; Xiao et al., 2016) (**Figure 1**).

### Characteristics of Included Studies

Twelve studies were published in Chinese (Zhang et al., 2003; Yu et al., 2005; Wang and Li, 2007; Luo et al., 2008; Liu et al., 2009; Luo et al., 2010; He and Li, 2011; Wang, 2012; Zhou et al., 2012; Liu et al., 2014; Tian et al., 2015; Xiao et al., 2016) and three studies in English (Zhang et al., 2006; Chen et al., 2011; Gui et al., 2015) between 2006 and 2015. Fourteen studies used male BALB/C mice (Zhang et al., 2003; Yu et al., 2005; Zhang et al., 2006; Wang and Li, 2007; Luo et al., 2008; Liu et al., 2009; Luo et al., 2010; Chen et al., 2011; He and Li, 2011; Wang, 2012; Zhou et al., 2012; Liu et al., 2014; Gui et al., 2015; Tian et al., 2015) and one used male Kunming mice (Xiao et al., 2016). Eight studies used mice weighing 12–16 g for experiments (Zhang et al., 2003; Yu et al., 2005; Luo et al., 2008; Liu et al., 2009; Luo et al., 2010; He and Li, 2011; Liu et al., 2014; Tian et al., 2015), one used mice weighing 12–14 g (Wang and Li, 2007), three used mice weighing 14–16 g (Zhang et al., 2006; Wang, 2012; Zhou et al., 2012), one used mice weighing 18–22 g (Xiao et al., 2016) and two did not provide information on mouse weight (Chen et al., 2011; Gui et al., 2015). VM models were established by intraperitoneal injection of CVB3 in all studies. In terms of AS-IV dosage, one study utilized 4.36 g/kg/d (Xiao et al., 2016), three utilized 600 mg/kg/d (Luo et al., 2008; Wang, 2012; Tian et al., 2015), one utilized 300 mg/kg/d (Chen et al., 2011), one utilized 120 mg/kg/d (Zhang et al., 2006), one utilized 40 mg/kg/d (Gui et al., 2015), one utilized 1 mg/kg/d (Wang and Li, 2007), two utilized 0.6 mg/kg/d (Luo et al., 2010; He and Li, 2011), and five did not specify the dose used (Zhang et al., 2003; Yu et al., 2005; Liu et al., 2009; Zhou et al., 2012; Liu et al., 2014). Twelve studies utilized mortality rate as outcome measure (Zhang et al., 2003; Yu et al., 2005; Zhang et al., 2006; Wang and Li, 2007; Luo et al., 2008; Liu et al., 2009; Chen et al., 2011; He and Li, 2011; Wang, 2012; Liu et al., 2014; Gui et al., 2015; Tian et al., 2015) and histopathological changes of myocardium were assessed in nine studies (Yu et al., 2005; Wang and Li, 2007; Luo et al., 2008; Liu et al., 2009; Wang, 2012; Zhou et al., 2012; Liu et al., 2014; Gui et al., 2015; Tian et al., 2015). Left ventricular ejection fraction (LVEF) was reported in one study (Chen et al., 2011), shortening fraction (FS) in two studies (Chen et al., 2011; Gui et al., 2015) and left ventricular endsystolic dimensions (LVEDd) in two studies (Chen et al., 2011; Gui et al., 2015), but the level of ST-segment depression was not mentioned. The cardiac troponin I (cTnI) level was reported in one study (Gui et al., 2015), creatine kinase (CK) in one study (Gui et al., 2015), superoxide dismutase (SOD) in two studies (Wang and Li, 2007; Luo et al., 2008), glutathione (GSH) in two studies (Wang and Li, 2007; Luo et al., 2008), reative oxygen species (ROS) in one study (Wang and Li, 2007), tumor necrosis factor-α (TNF-α) in two studies (Zhou et al., 2012; Gui et al., 2015), myocardial apoptosis index in two studies (Zhang et al., 2003; Luo et al., 2010), B-cell lymphoma-2 (Bcl-2) in two study (Luo et al., 2010), Bcl2-associated X (Bax) in one study (Luo et al., 2010), and nuclear factor κB (NF-κB) in one study (Gui et al., 2015). The characteristics of the included studies are summarized in **Table 1**.

**Table 1:** Characteristics of the 15 included studies.

Study (years)	Species (Sex; n = experimental/control group)	Weight	Model (method)	Treatment group (Method to astragal sides)	Control group	Outcome Index (time)	Intergroup Differences
Zhang et al. (2003)	BALB/C mice (male; 18/18)	12-16g	By intraperitoneal injection of EMEM solution containing 1×10^^9^ TCID50 CVB3 (0.1ml) at the age of 4 weeks	By intragastric administration of 9% AS-IV (0.1ml; qd) for 7d after establishing model	By intragastric administration of normal saline (0.1ml; qd) for 7d after establishing model	1.Mortality rate 2.CVF 3.PIP 4. PIIINP 5.Myocardial apoptosis index	1.P < 0.05 2.P < 0.01 3.P < 0.01 4.P < 0.01 5. P < 0.01
Yu et al. (2005)	BALB/C mice (male; 20/20)	12-16g	By intraperitoneal injection of CVB3 at the age of 4 weeks	By intragastric administration of 9% AS-IV (0.1ml; qd) for 7d after establishing model	By intragastric administration of sodium carboxymethyl cellulose solution (0.1ml; qd) for 7d after establishing model	1.Mortality rate 2.Histopathological changes of myocardium	1.P < 0.05 2.P < 0.01
Zhang et al. (2006)	BALB/C Mice (male; 15/16)	14-16g	By intraperitoneal injection of CVB3 at the age of 4 weeks	By intragastric administration of AS-IV (120mg/kg; qd) for 7d after establishing model	By intragastric administration of isometric normal saline solution (qd) for 7d after establishing model	1.Mortality rate 2.HW/BW ratios 3.Virus titers of hearts 4.Scores of necrosis and infiltration	1.P < 0.01 2.P < 0.01 3.P < 0.01 4.P < 0.01
Wang and Li (2007)	BALB/C Mice (male; 30/30)	12-14g	By intraperitoneal injection of RPIM-1640 solution containing 1 ×10^^5^ TCID50 CVB3 at the age of 4 weeks	By intragastric administration of AS-IV (1mg/kg; qd) for 7d after establishing model	By intragastric administration of isometric sodium carboxymethyl cellulose solution (qd) for 7d after establishing model	1.Mortality rate 2.Histopathological changes of myocardium 3.T-SOD 4.GSH-PX 5.CAT 6.MPO 7.ROS	1.P < 0.01 2.P < 0.05 3.P < 0.01 4.P < 0.01 5.P < 0.01 6.P < 0.01 7.P < 0.01
Luo et al. (2008)	BALB/C mice (male; 10/10)	12-16g	By intraperitoneal injection of EMEM solution containing 1×10^^2^ TCID50 CVB3 (0.1 ml) at the age of 4 weeks	By intragastric administration of 9% AS-IV (600mg/kg) (0.1ml; qd) for 7d after establishing model	By intragastric administration of normal saline (0.1ml; qd) for 7d after establishing model	1.Mortality rate 2.Histopathological changes of myocardium 3.T-SOD 4.GSH-PX 5.CAT	1.P < 0.05 2.P < 0.01 3.P < 0.05 4.P < 0.05 5.P < 0.05
Liu et al. (2009)	BALB/C mice (male; 15/15)	12-16g	By intraperitoneal injection of virus culture medium containing 1 ×10^^2^ TCID50 CVB3 (0.1ml) at the age of 4 weeks	By intragastric administration of 9% AS-IV (0.1ml; qd) for 14d after establishing model	By intragastric administration of sodium carboxymethyl cellulose solution (0.1ml; qd) for 14d after establishing model	1.Mortality rate 2.Histopathological changes of myocardium 3.MIF protein	1.P < 0.05 2.P < 0.01 3.P < 0.01
Luo et al. (2010)	BALB/C mice (male; 10/10)	12-16g	By intraperitoneal injection of EMEM solution containing 1 ×10^^5^ TCID50 CVB3 (0.1ml) at the age of 4 weeks	By intragastric administration of 9% AS-IV (0.6mg/kg) (0.1ml; qd) for 7d after establishing model	By intragastric administration of normal saline (0.1ml; qd) for 7d after establishing model	1.Myocardial apoptosis index 2.Bcl-2 3.Bax	1.P < 0.01 2.P < 0.01 3.P < 0.01
Chen et al. (2011)	BALB/C mice (male; 20/30)	NM	By intraperitoneal injection of CVB3 (100ul) at the age of 4 weeks and treatments monthly for 9 month to induce dilated cardiomyopathy	By intragastric administration of AS-IV (300mg/L; qd) containing drinking water after establishing model	By intragastric administration of isometric plain drinking water (qd) after establishing model	1.Mortality rate 2.LVEDd 3.LVEDs 4.LVEF 5.FS 6.PICP 7.PINP 8.PICP/PINP ratio 9.TGF-β1 10.pSmad2/3 11.Smad4 12.Smad7	1.P < 0.05 2.P < 0.001 3.P < 0.001 4.P < 0.001 5.P < 0.001 6.P < 0.05 7.p > 0.05 8.P < 0.01 9.P < 0.05 10.P < 0.05 11.P < 0.01 12.p > 0.05
He and Li (2011)	BALB/C mice (male; 15/15)	12-16g	By intraperitoneal injection of Eagle’ s culture media containing 1 ×10^^2^ TCID50 CVB3 (0.1 ml) at the age of 4 weeks	By intragastric administration of 9% AS-IV (0.6mg/kg; qd) for 15d after establishing model	By intragastric administration of isometric hydroxymethyl cellulose sodium solution (qd) for 15d after establishing model	1.IGF-1 in plasma 2.IGF-1 Protein in myocardium 3.IGF-1R Protein in myocardium 4.IGFBP3 Protein in myocardium	1.P < 0.05 2.P < 0.05 3.P < 0.05 4.P < 0.05
Wang (2012)	BALB/C mice (male; 30/30)	14-16g	By intraperitoneal injection of CVB3 (0.1ml) at the age of 4 weeks	By intragastric administration of AS-IV (0.1ml; qd) for 7d after establishing model	By intragastric administration of normal saline (0.1ml; qd) for 7d after establishing model	1.Histopathological changes of myocardium 2.P38MAPK	1.P < 0.05 2.P < 0.05
Zhou et al. (2012)	BALB/C mice (male; 30/30)	14-16g	By intraperitoneal injection of 1 ×10^^2^ TCID50 CVB3 (0.1ml) at the age of 4 weeks	By intragastric administration of AS-IV (0.1ml; qd) for 7d after establishing model	By intragastric administration of normal saline (0.1ml; qd) for 7d after establishing model	1.Histopathological changes of myocardium 2.TNF-α	1.P < 0.05 2.P < 0.05
Liu et al. (2014)	BALB/C mice (male; 20/20)	12-16g	By intraperitoneal injection of virus culture medium containing 1 ×10^^2^ TCID50 CVB3 (0.1ml) at the age of 4 weeks	By intragastric administration of 9% AS-IV (0.1ml; qd) for 14d after establishing model	By intragastric administration of sodium carboxymethyl cellulose solution (0.1ml; qd) for 14d after establishing model	1.Mortality rate 2.Histopathological changes of myocardium 3.Th17 4.IL-23 5.IL-17	1.P > 0.003 2.P < 0.05 3.P < 0.05 4.P < 0.05 5.P < 0.05
Gui et al. (2015)	BALB/C mice (male; 15/15)	NM	By intraperitoneal injection of 1 ×10^^3^ TCID50 CVB3 at the age of 6 weeks	By intragastric injection of AS-IV (40mg/kg; qd) for 7d after establishing model	By intragastric injection of isometric normal saline (qd) for 7d after establishing model	1.The changes of body weight 2.Mortality rate 3.CK-MB 4.cTnI 5.Myocarditis score 6. PWd 7.SWd 8.LVEDd 9.LVEDs 10.EF 11.FS 12.HR 13.TNF-α in myocardium 14.IL-1β in myocardium 15.IL-6 in myocardium 16.MCP-1 in myocardium 17.TNF-α in serum 18.IL-1β in serum 19.IL-6 in serum 20.MCP-1 in serum 21.CD3 22.CD11b 23.NF-κB DNA 24.A20	1.P < 0.01 2.P < 0.01 3.P < 0.01 4.P < 0.01 5.P < 0.01 6.P < 0.01 7.p > 0.05 8.P < 0.01 9.p < 0.01 10.P < 0.01 11.P < 0.01 12.P > 0.05 13.P < 0.001 14.P < 0.01 15.P < 0.001 16.P < 0.001 17.P < 0.001 18.P < 0.001 19.P < 0.001 20.P < 0.001 21.P < 0.001 22.P < 0.01 23.P < 0.01 24.P < 0.001
Tian et al. (2015)	BALB/C mice (male; 20/20)	12-16g	By intraperitoneal injection of Eagle’ s culture media containing 1 ×10^^2^ TCID50 CVB3 (0.1 ml) at the age of 4 weeks	By intragastric administration of 9% AS-IV (600mg/kg; 0.1ml; qd) for 7d after establishing model	By intragastric administration of sodium carboxymethyl cellulose solution (0.1ml; qd) for 7d after establishing model	1.Mortality rate 2.Histopathological changes of myocardium 3.TL1A protein	1.P < 0.05 2.P < 0.01 3.P < 0.01
Xiao et al. (2016)	Kunming mice (male; 15/15)	18-22g	By intraperitoneal injection of CVB3 (0.1ml; qod) for 4 times	By intragastric administration of AS-IV (4.36g/kg; qd) for 15d after establishing model	By intragastric administration of isometric 0.5% sodium carboxymethyl cellulose solution (20ml/kg; qd) for 15d after establishing model	1.TIMP-1 2.MMP-1	1.P < 0.01 2.P < 0.01

A20, TNF-α induced protein 3; AS-IV, astragaloside IV; Bax, Bcl2-associated X; Bcl-2, B-cell lymphoma-2; CAT, catalase; cTnI, cardiac troponin I; CD3, clusters of differentiation 3; CD11b, clusters of differentiation 11b; CVB3, coxsackievirus B3; CVF, collagen volume fraction; EF, ejection fraction; EMEM, Eagle’s minimal essential medium; FS, shortening fraction; GSH-PX, glutathion peroxidase; HR, heart rate; HW/BW, heart weight/body weight; IGF-1, insulin-like growth factor-1; IGF-1R, insulin-like growth factor-1R; IGFBP3, Insulin-like growth factor binding protein 3; IL-1β, interleukin-1β; IL-6, interleukin-6; IL-17, interleukin-17; IL-23, interleukin-23; LVEDd, left ventricular end-diastolic dimensions; LVEDs, left ventricular endsystolic dimensions; LVEF, left ventricular ejection fraction; MCP-1, monocyte chemoattractant protein-1; MIF, macrophage migration inhibitory factor; MMP-1, matrix metalloproteinase-1; MPO, myeloperoxidase; NF-κB, nuclear factor-κB; P38MAPK, P38 mitogen-activated protein kinase; PICP, carboxyterminal propeptide of type I procollagen; PINP, aminoterminal propeptide of type I procollagen; PIP, propeptide of procollagen type I; PIIINP, procollagen type III aminoterminal peptide; pSmad2/3, phosphorylated small mothers against decapentaplegic 2/3; PWd, posterior wall thickness in end diastole; RPIM-1640, Roswell Park Memorials Institute 1640; ROS, reative oxygen species; Smad4, drosophila mothers against decapentaplegic 4; Smad7, drosophila mothers against decapentaplegic7; SWd, septal wall thickness; TCID50, tissue culture infectious dose 50; TGF-β1, transforming growth factor-β1; Th17, helper T cell 17; TIMP-1, tissue inhibitor of metalloproteinase-1; TL1A, TNF-like ligand 1 aberrance; TNF-α, tumor necrosis factor-α; T-SOD, total superoxide dimutase.

### Study Quality

The quality of all the included studies was evaluated and scored from 2 to 5. Two studies employed methods of random allocation (Zhang et al., 2006; Liu et al., 2014). Four studies specified the time of model induction before random allocation (Zhang et al., 2003; Wang and Li, 2007; Chen et al., 2011; Gui et al., 2015). Allocation to different groups was not concealed in any of the studies. Two studies declared compliance with animal welfare regulations (Zhang et al., 2006; Gui et al., 2015). However, no studies reported blinding of caregivers and the methods of random outcome assessment. One study described blinding of outcome assessor (Wang, 2012). All studies specified complete outcome data and no other sources of bias. Nine studies described the free of selective outcome reporting (Zhang et al., 2003; Yu et al., 2005; Zhang et al., 2006; Liu et al., 2009; Chen et al., 2011; Liu et al., 2014; Gui et al., 2015; Tian et al., 2015; Xiao et al., 2016). The methodological quality is summarized in **Table 2**.

**Table 2:** Quality assessment of included studies.

Study	A	B	C	D	E	F	G	H	I	J	Total
Zhang et al. (2003)	?	**+**	**–**	**–**	**–**	**–**	**–**	**+**	**+**	**+**	4
Yu et al. (2005)	**–**	**–**	**–**	**–**	**–**	**–**	**–**	**+**	**+**	**+**	3
Zhang et al. (2006)	**+**	**–**	**–**	**+**	**–**	**–**	**–**	**+**	**+**	**+**	5
Wang and Li (2007)	?	**+**	**–**	**–**	**–**	**–**	**–**	**+**	?	**+**	3
Luo et al. (2008)	?	**–**	**–**	**–**	**–**	**–**	**–**	**+**	?	**+**	2
Liu et al. (2009)	?	**–**	**–**	**–**	**–**	**–**	**–**	**+**	**+**	**+**	3
Luo et al. (2010)	?	**–**	**–**	**–**	**–**	**–**	**–**	**+**	?	**+**	2
Chen et al. (2011)	?	**+**	**–**	**–**	**–**	**–**	**–**	**+**	**+**	**+**	4
He and Li (2011)	?	**–**	**–**	**–**	**–**	**–**	**–**	**+**	?	**+**	2
Wang (2012)	?	**–**	**–**	**–**	**–**	?	**+**	**+**	?	**+**	3
Zhou et al. (2012)	?	**–**	**–**	**–**	**–**	**–**	**–**	**+**	?	**+**	2
Liu et al. (2014)	**+**	**–**	**–**	**–**	**–**	**–**	**–**	**+**	**+**	**+**	4
Gui et al. (2015)	**–**	**+**	**–**	**+**	**–**	**–**	**–**	**+**	**+**	**+**	5
Tian et al. (2015)	?	**–**	**–**	**–**	**–**	**–**	**–**	**+**	**+**	**+**	3
Xiao et al. (2016)	?	**–**	**–**	**–**	**–**	**–**	**–**	**+**	**+**	**+**	3

sequence generation; B: baseline characteristics; C: allocation concealment; D: random housing and animal welfare; E: blinding of caregivers and/or investigators; F: random outcome assessment; G: blinding of outcome assessor; H: complete outcome data; I: selective outcome reporting; J: other sources of bias. “+” indicates low risk of bias; “***-***” indicates high risk of bias; and “?” indicates an unclear risk of bias.

### Effectiveness

#### Mortality

**Figure 2:**
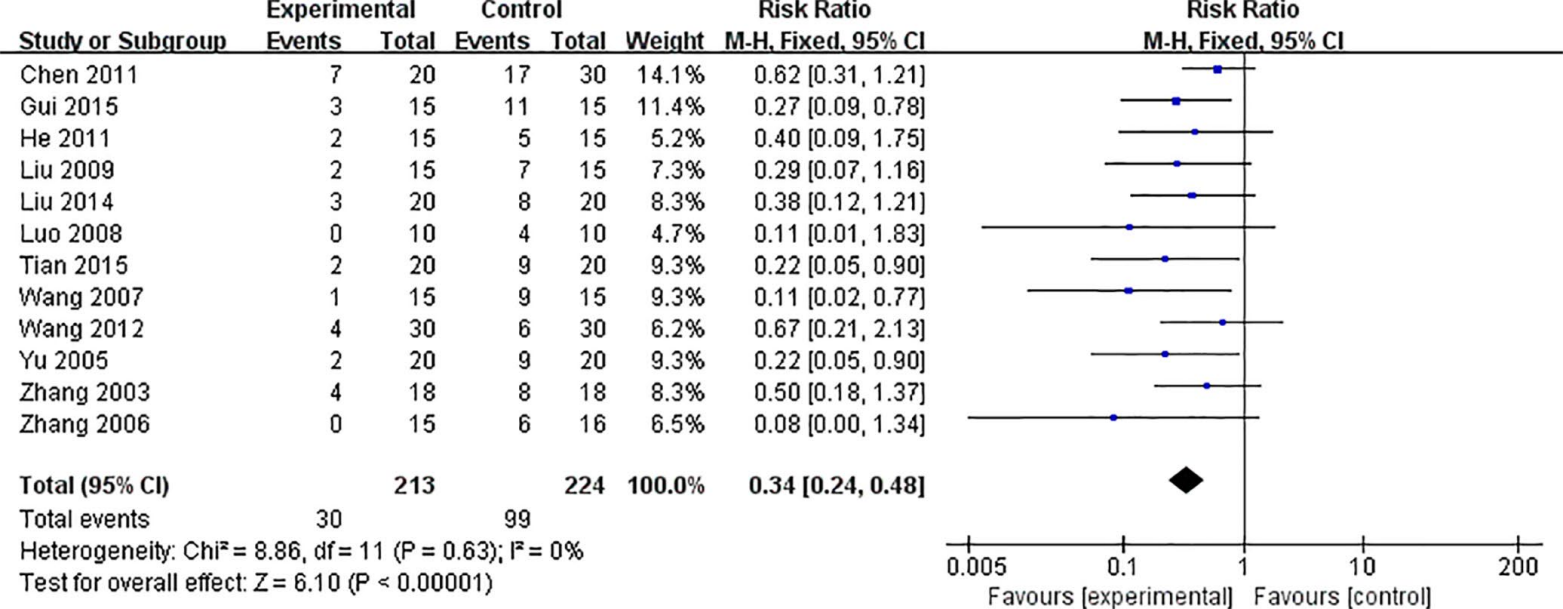
The forest plot: effects of AS-IV for decreasing mortality compared with control group.

Meta-analysis of 12 studies (Zhang et al., 2003; Yu et al., 2005; Zhang et al., 2006; Wang and Li, 2007; Luo et al., 2008; Liu et al., 2009; Chen et al., 2011; He and Li, 2011; Wang, 2012; Liu et al., 2014; Gui et al., 2015; Tian et al., 2015) showed that AS-IV induces a significant reduction in mortality of VM animals, compared with control [n = 437, RR 0.34, 95% CI (0.24 to 0.48), P < 0.0001; heterogeneity: χ^2^ = 8.86, I^2^ = 0%] (**Figure 2**).

#### Histopathological Changes of Myocardium

**Figure 3:**
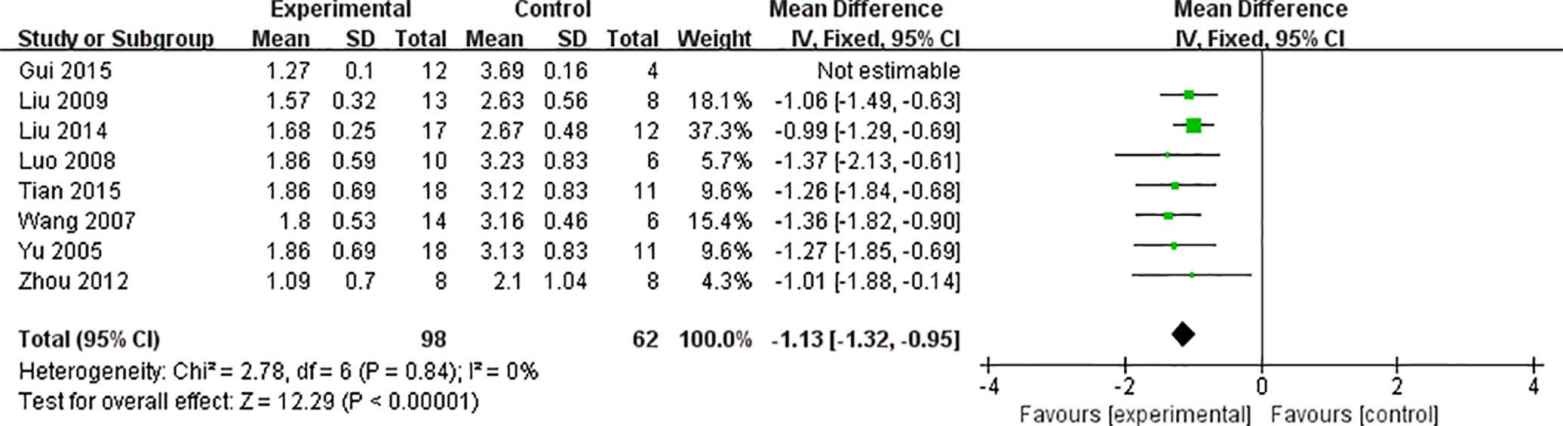
The forest plot: effects of AS-IV for decreasing the inflammation of myocardium and pathological score compared with control group.

Meta-analysis of eight studies (Yu et al., 2005; Wang and Li, 2007; Luo et al., 2008; Liu et al., 2009; Zhou et al., 2012; Liu et al., 2014; Gui et al., 2015; Tian et al., 2015) revealed significant effects of AS-IV on reducing inflammation of the myocardium and pathological score in VM animals, compared with control [n = 176, MD -1.83 95% CI (-1.95 to -1.71), P < 0.0001; heterogeneity: χ^2^ = 107.56, I^2^ = 93%]. After sensitivity analyses, we removed one study (Gui et al., 2015) that modeling in mice at the age of 6 weeks. Meta-analysis of seven studies showed significant effects of AS-IV on reducing myocardial inflammation and pathological scores in VM animals [n = 160, MD -1.13 95% CI (-1.32 to -0.95), P < 0.0001; heterogeneity: χ^2^ = 2.78, I^2^ = 0%] (**Figure 3**).

#### LVEF

**Figure 4:**
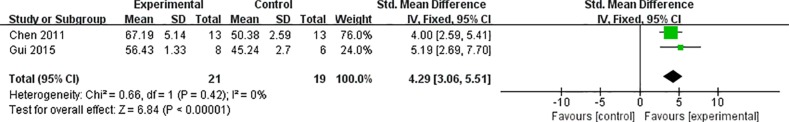
The forest plot: effects of AS-IV for improving LVEF compared with control group.

Data from meta-analysis of two studies (Chen et al., 2011; Gui et al., 2015) showed that AS-IV treatment induced significant improvement of LVEF in animals with VM [n = 40 SMD 4.29 95% CI (3.06 to 5.51), P < 0.0001; heterogeneity: χ^2^ = 0.66, I^2^ = 0%] (**Figure 4**).

#### LVEDs

**Figure 5:**
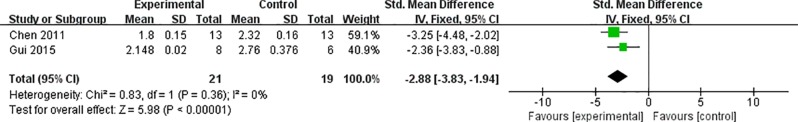
The forest plot: effects of AS-IV for decreasing LVEDds compared with control group.

Data from meta-analysis of two studies (Chen et al., 2011; Gui et al., 2015) revealed significant suppressive effects of AS-IV on LVEDs of VM animals [n = 40 SMD -2.88 95% CI (-3.83 to -1.94), P < 0.0001; heterogeneity: χ^2^ = 0.83, I^2^ = 0%] (**Figure 5**).

#### Cardiac Enzymes and/or Troponin

One study (Gui et al., 2015) reported significant reduction CK-MB and CTnI levels of animals with VM treated with AS-IV (P < 0.05).

#### Cardioprotective Mechanisms

**Figure 6:**
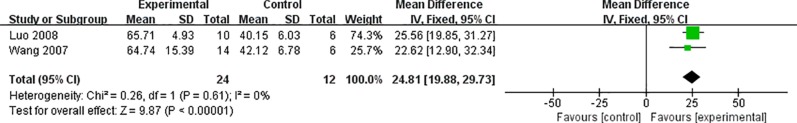
The forest plot: effects of AS-IV for increasing the T-SOD compared with control group.

**Figure 7:**
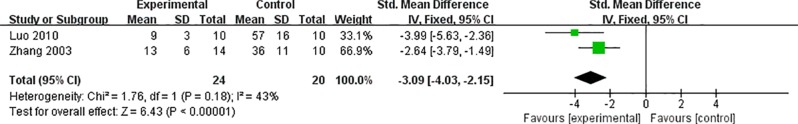
The forest plot: effects of AS-IV for reducing apoptosis index compared with control group.

**Figure 8:**
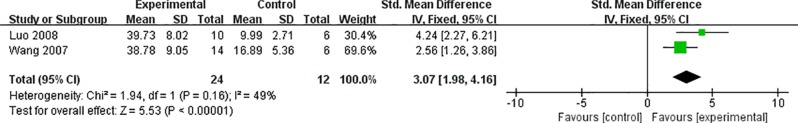
The forest plot: effects of AS-IV for increasing GSH compared with control group.

**Figure 9:**
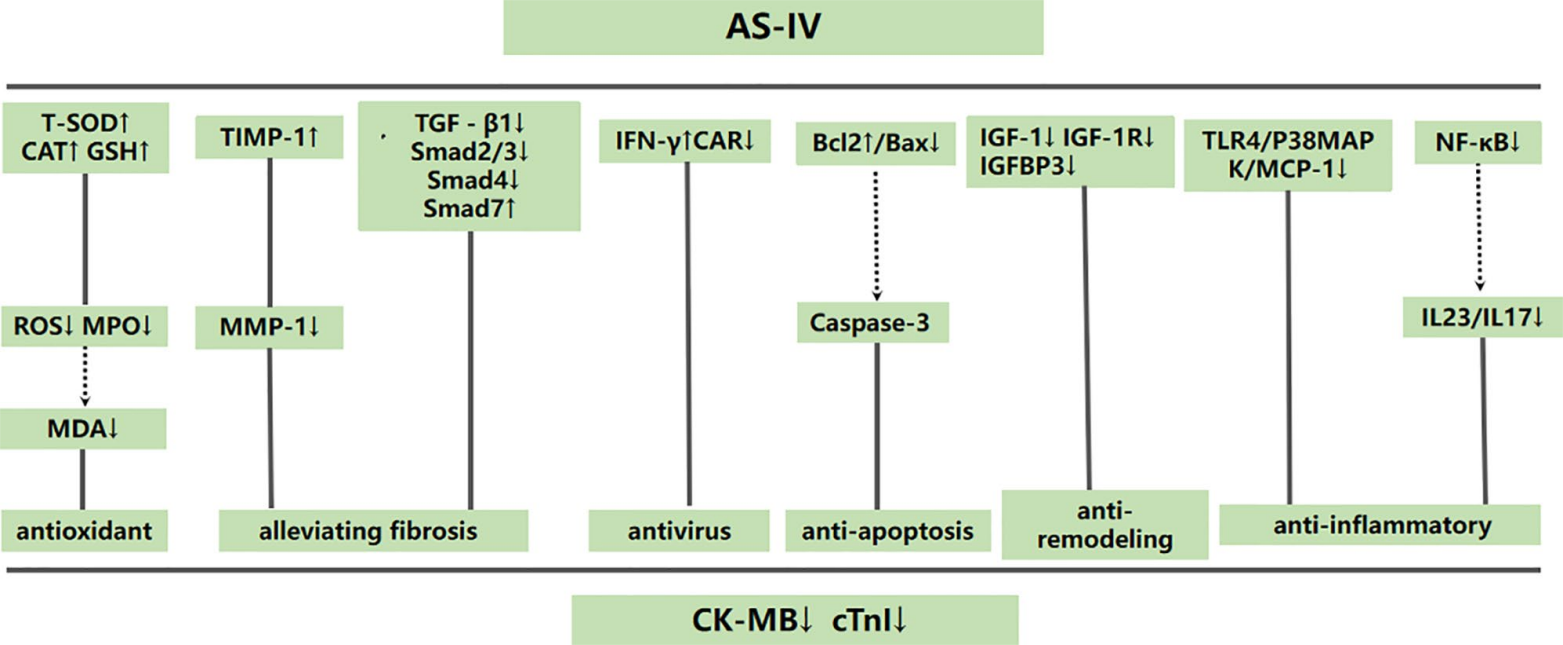
A schematic representation of cardioprotective mechanisms of astragaloside IV for viral myocarditis. Solid lines indicate established effects, whereas dashed lines represent putative mechanisms.

Two studies (Wang and Li, 2007; Luo et al., 2008) reported a significant increase in T-SOD of VM animals by AS-IV, compared with the control group [n = 36 MD 24.81 95% CI (19.88 to 29.73), P < 0.0001; heterogeneity: χ^2^ = 0.26, I^2^ = 0%] (**Figure 6**). Two studies (Zhang et al., 2003; Luo et al., 2010) focused on AS-IV-induced reduction of apoptosis index [n = 44 SMD -3.09 95% CI (-4.03 to -2.15), P < 0.0001; heterogeneity: χ^2^ = 1.76, I^2^ = 43%] (**Figure 7**), two (Wang and Li, 2007; Luo et al., 2008) on AS-IV-induced increase in GSH [n = 36 SMD 3.07 95% CI (1.98 to 4.16), P < 0.0001; heterogeneity: χ^2^ = 1.94, I^2^ = 49%] (**Figure 8**), two (Zhou et al., 2012; Gui et al., 2015) on AS-IV-mediated reduction of TNF-α (P < 0.05), one (Wang and Li, 2007) on reduction of ROS levels by AS-IV, one (Luo et al., 2010) on AS-IV-mediated increase in Bcl-2 (P < 0.05) and reduction of Bax (P < 0.05), one (Gui et al., 2015) on AS-IV-triggered NF-κB (P < 0.05), and one (Gui et al., 2015) on reduction of IL-6 by AS-IV (P < 0.05). A schematic representation of the cardioprotective mechanisms of AS-IV is summarized in **Figure 9**.

## Discussion

### Summary of Evidence

To our knowledge, this is the first preclinical systematic review (including 15 *in vivo* studies on a total of 577 animals) focused on evaluating the efficacy of AS-IV against VM and its potential mechanisms of action. Our findings clearly support cardioprotective effects of AS-IV in animal models of VM, mainly through improving anti-remodeling of the myocardium, anti-inflammatory, anti-apoptosis, anti-virus, antioxidant, and alleviation of myocardial fibrosis.

### Methodological Considerations

Assessing of the validity of the included studies is a key component of a systematic review, and subsequently affects its analysis, interpretation and conclusions. Based on the Cochrane Handbook for Systematic Reviews of Interventions (Higgins and Green, 2011), the validity of an individual study should include two dimensions: external validity and internal validity. The former refers to whether the study is asking the appropriate research questions and the latter is in a manner free from the risk of bias of the included studies. In the present study, an appropriate research question was initially set up to evaluate the efficacy and possible mechanism of AS-IV in the treatment of the animals with VM using preclinical systematic review method. When assessments of methodological quality of included preclinical studies, two main risk of bias tools have been developed, including the Collaborative Approach to Meta-Analysis and Review of Animal Data from Experimental Studies (CAMARADES) 10-item checklist (Van et al., 2007) and the SYstematic Review Centre for Laboratory animal Experimentation (SYRCLE)’s Risk of Bias tool (Hooijmans et al., 2014). The CAMARADES 10-item checklist is specific for stroke and neuroprotective study on animal model while the SYRCLE’s Risk of Bias tool is extensively used to assess the methodological quality of animal studies. Here, we selected the SYRCLE’s Risk of Bias tool to assess the quality of included study and the result indicated that the quality scores of all studies were generally moderate. Thus, we should treat the present results cautiously when applying the study’s findings.

Accumulating evidence demonstrates that animal reports on biomedical research are often inadequate in many areas (Kilkenny et al., 2010). An earlier survey evaluated 271 randomly selected articles about animal reports by the National Centre for the Replacement, Refinement and Reduction of Animals in Research (NC3Rs). The results showed that 59% articles overall declared the hypotheses or objectives of the study and the characteristics and number of animals used. Randomization (87%) or blindness (86%) was not used in the majority of the articles to reduce bias of animal selection and outcome assessments. In total, 70% of the articles used statistical methods to fully describe publications and present the results in an accurate manner (Kilkenny et al., 2009). Similarly, the articles selected for our analysis had methodological deficiencies, such as seldom using the blind method and allocation concealment. Therefore, concerns of poor experimental design and lack of transparent reports are raised, which can lead to failure of translation of preclinical animal research into clinical treatments for human diseases (Baker et al., 2014). Reporting guidelines develop clear and standard checklist formats to make animal reports more complete and transparent, and thus increase the value of animal reports in clinical practice (Moher et al., 2015). The Animal Research: Reporting In Vivo Experiments (ARRIVE) guidelines, which provide a checklist of 20 items to promote high-quality, comprehensive reporting, have been accepted by over 300 research journals worldwide (Kilkenny et al., 2010). We recommend adherence to these guidelines for the further design of animal studies. In particular, attention should be paid to calculation of sample size, blinding of outcome assessor and blinding of caregivers (Macleod et al., 2004). As a minimum requirement, harmonized animal research reporting principles (HARRP) has been set up based on a comparative analysis of the ARRIVE guidelines by the Gold Standard Publication Checklist (GSPC) and the Institute of Laboratory Animal Research (ILAR) Guidance. Thus, the HARRP provided the minimal reporting standards for animal-based research (Osborne et al., 2018).

### Implications

This study confirmed that AS-IV induces a significant reduction in mortality, improves myocardial pathology, and suppresses expression of cardiac enzymes, supporting its utility as a therapeutic option for patients with VM. However, translation of preclinical experiments remains challenging in predicting the effectiveness of therapeutic strategies in clinical trials (Hackam, 2007). The differences in drug doses and time of drug administration between humans and animals are considered the main reasons underlying failure of translation of research from bench to patient bedside (Baker et al., 2014). It is important to note that the 15 articles included in this study are not consistent in terms of drug dose and time of drug administration. Thus, we recommend establishing drug concentration gradients and grouping administration times to establish the optimal treatment strategy. In view of the differences between clinical trials and animal research, further high-quality randomized controlled trials of AS-IV for VM are required.

Several methods are commonly used for establishing animal models of myocarditis. (1) CVB3 or encephalomyocarditis virus (EMCV) injection: the advantages of this model is use of virus, which is close to clinical practice and suitable for studying CVB3 replication. However, this method is associated with high mortality rates and requires high biosafety standards. (2) Reovirus or murine adenovirus type 1 (MAV-1) injection is a unique model of pediatric myocarditis but is not clinically relevant. (3) *Trypanosoma cruzi* (*T. cruzi*) infection model, which could recapitulate the course of Chagas disease, but requires a long period for study. (4) Immunization with α-myosin heavy chain (α-MyHC) or troponin I peptide and complete Freund’s adjuvant (CFA). The limitation of this model, which is suitable for studying transition from myocarditis to DCM, is non-physiological disease induction. (5) T cell receptor specific to α-MyHC (TCR-M) transgenic mice. The advantages of this model are biosafety and suitability for studying the pathophysiology of heart-specific T cells. However, the lack of non-specific T cells presents a drawback. (6) Programmed cell death protein-1 (PD-1)/PD-1 ligand (PD-L1) deficiency. This model is suitable for investigating the side-effects of anti-PD-1/PD-1L therapy but involves multiple organs. (7) A human leukocyte antigen (HLA-DQ8) transgenic mouse model is beneficial for studying cardiac antigen presentation but a human-mouse chimeric system is lacking at present (Błyszczuk, 2019). All the studies included in this review utilized a myocardial model induced by CVB3 injection. Since CVB3 is closely related to the pathogenesis of myocarditis in human, this method of modeling is rational. We therefore recommend application of CVB3 to establish models of VM.

### Mechanisms

Based on the findings of the included studies, the possible mechanisms of AS-IV mediated cardiovascular protection are as follows: (1) antioxidant through increasing T-SOD, GSH-PX and CAT levels (Wang and Li, 2007; Luo et al., 2008) to inhibit release of ROS and MPO (Wang and Li, 2007), (2) anti-inflammatory activity through suppression the TLR4/P38MAPK/MCP-1 (Wang and Li, 2007; Zhou et al., 2012) and IL23/IL17 pathways (Liu et al., 2014) and inhibition of NF-κB (Zhou et al., 2012) as well as MIF (Liu et al., 2009) and A20 (Liu et al., 2015), (3) alleviation of myocardial fibrosis through inhibiting TGF-β1, Smad2/3 and Smad4 and enhancing Smad7 (Chen et al., 2011) and increasing TIMP-1to reduce MMP-1 release (Xiao et al., 2016), (4) prevention of viral spread through increasing IFN-γ (Zhang et al., 2006) and decreasing CAR expression (Yu et al., 2005), (5) inhibition of apoptosis through enhancing the Bcl-2/Bax ratio (Luo et al., 2010), (6) anti-remodeling of myocardium through suppressing the expression of IGF-1, IGF-1R and IGFBP3 (He and Li, 2011).

## Conclusion

The collective research clearly demonstrates that AS-IV decreases mortality, inflammation of the myocardium, pathological score and cardiac enzyme activity and improves heart function. Moreover, AS-IV exerts potential cardioprotective function of in VM primarily *via* improving anti-remodeling of myocardium, anti-inflammatory, anti-apoptosis, anti-virus, antioxidant and alleviation of myocardial fibrosis. Thus, AS-IV is a cardioprotective recruit for further clinical trials on VM.

## Author Contributions

Study conception and design: ZZ, G-QZ, and YW. Acquisition and analysis of data: ZZ, Z-HW, QZ, and L-HD. Manuscript writing: ZZ, Z-HW, and G-QZ. All authors gave final approval.

## Funding

This project was supported by the grant of National Natural Science Foundation of China (81473491/81573750/81173395/H2902).

## Conflict of Interest

The authors declare that the research was conducted in the absence of any commercial or financial relationships that could be construed as a potential conflict of interest.
